# RIPC Protects Against Renal Ischaemia–Reperfusion Injury by Activating SIRT3/Parkin‐Mediated Mitophagy

**DOI:** 10.1111/jcmm.71372

**Published:** 2026-10-11

**Authors:** Qian Cui, Lina Zhao, Wenjun Dai, Shuyan Sun, Yinan Li, Chenghui Zhou, Hanjun Pei

**Affiliations:** ^1^ Department of Respiratory and Critical Care Medicine The First Affiliated Hospital of Baotou Medical College Baotou China; ^2^ Department of Critical Care Medicine Tianjin Medical University General Hospital Tianjin China; ^3^ Department of Cardiology International Mongolian Hospital of Inner Mongolia Hohhot China; ^4^ Department of Cardiology The First Affiliated Hospital of Baotou Medical College Baotou China; ^5^ Department of Anesthesiology Beijing Luhe Hospital, Capital Medical University Beijing China; ^6^ Department of Intensive Care Medicine Beijing Luhe Hospital, Capital Medical University Beijing China

**Keywords:** acute kidney injury, ischaemia reperfusion, mitophagy, remote ischaemic preconditioning, SIRT3

## Abstract

Remote ischaemic preconditioning (RIPC) protects against renal ischaemia–reperfusion injury (IRI), yet its precise mechanism remains incompletely understood, particularly regarding the role of mitochondrial quality control. This study investigated whether SIRT3‐dependent mitophagy mediates the protective effects of RIPC against acute kidney injury (AKI) induced by IRI and further examined the integrity of mitophagic flux and involvement of the classic Parkin‐mediated mitophagic pathway. We used a murine bilateral renal ischaemia–reperfusion injury (IRI) model, including RIPC pretreatment, 3‐methyladenine (3‐MA) intervention and SIRT3 knockout intervention, and a HK‐2 cell hypoxia/reoxygenation (H/R) model, including SIRT3 siRNA knockdown, SIRT3 overexpression and chloroquine (CQ) intervention. Renal function, histopathological changes, apoptosis, mitochondrial membrane potential and mitophagy were assessed using biochemical analysis, histological and TUNEL staining, JC‐1 staining, transmission electron microscopy, mitochondrial protein analysis and immunofluorescence colocalization of Parkin and LC3B. RIPC improved renal function, alleviated tubular injury and apoptosis, and enhanced mitophagy in mice, which was attenuated by SIRT3 knockout or 3‐MA. In HK‐2 cells, RIPC plasma protected against H/R injury and activated mitophagic flux, as verified by CQ treatment, while SIRT3 knockdown abolished these effects and SIRT3 overexpression maintained protective mitophagy levels. RIPC pretreatment restored the IRI‐induced reduction in Parkin and LC3B colocalization in renal tubular epithelial mitochondria, and this effect was attenuated by 3‐MA. RIPC alone had no detectable cytotoxicity or effect on basal mitophagy. In conclusion, RIPC mitigates renal IRI by activating SIRT3‐dependent mitophagy, with involvement of the Parkin‐mediated mitophagic pathway. Our in vivo and in vitro data support the central role of SIRT3 in RIPC‐induced renoprotection and provide evidence for an intact mitophagic flux mechanism.

## 1 Introduction

Acute kidney injury (AKI) following percutaneous coronary intervention (PCI), termed PCI‐AKI, is a critical complication associated with significant morbidity and mortality, and renal ischaemia–reperfusion injury (IRI) is its core pathological contributor [1]. Mitochondrial dysfunction acts as a central mediator in the pathogenesis of PCI‐AKI, which is driven by ischaemic insults, inflammatory cascades and tubular epithelial cell apoptosis [2]. As the primary energy source for renal tubular cells, mitochondria are particularly vulnerable to IRI [3], making mitochondrial quality control—specifically the selective clearance of damaged organelles via mitophagy—a promising therapeutic target for PCI‐AKI prevention [4]. Mitophagy—a selective form of autophagy that removes damaged mitochondria—maintains mitochondrial homeostasis and protects against renal IRI [5]. Preclinical studies have demonstrated that enhancing mitophagy alleviates AKI by reducing mitochondrial reactive oxygen species and cellular apoptosis [6]. These observations support mitophagy as a rational target for preventing PCI‐AKI.

Remote ischaemic preconditioning (RIPC), a non‐invasive intervention involving transient limb ischaemia–reperfusion cycles, has emerged as a potent endogenous renoprotective approach against renal IRI [7]. RIPC induces adaptive changes in pre‐treated tissues, including alterations in energy metabolism, improvement of electrolyte and acid–base balance, reduction of reactive oxygen species production, attenuation of inflammatory response and apoptosis, and enhancement of microcirculation perfusion [8, 9], which collectively increase the tolerance of renal tissues to subsequent IRI. Notably, RIPC has been shown to exert organ‐protective effects by inducing autophagy in multiple tissues, including the heart, liver and brain [10, 11, 12]. RIPC improves renal function and pathological injury in various AKI models (cisplatin, LPS and IRI), and preserves mitochondrial function in renal tubular epithelial cells [13], yet the specific molecular mechanism underlying RIPC‐regulated autophagy/mitophagy in renal IRI remains incompletely elucidated.

The sirtuin family (SIRT1‐7), NAD^+^‐dependent deacetylases regulating metabolism, ageing and stress responses [14], includes SIRT3 as a key mitochondrial guardian. Notably, RIPC has been shown to prevent spinal cord ischaemia–reperfusion injury in mice by activating the NMDAR/AMPK/PGC‐1α/SIRT3 signalling pathway [15], highlighting the conserved role of SIRT3 as a downstream effector of RIPC‐mediated organ protection across different tissues. The kidney, as one of the most energy‐consuming organs with high mitochondrial content, abundantly expresses SIRT3 [16], and SIRT3 has been identified as a critical regulator of mitophagy in renal IRI: SIRT3 knockout exacerbates proximal renal tubular damage under ischaemia–reperfusion conditions, while its overexpression protects renal tubular epithelial cells and alleviates IRI‐induced injury [17]. However, whether SIRT3 mediates RIPC‐induced mitophagy and subsequent renal protection against IRI, and the integrity of the mitophagic flux involved in this process, remains to be rigorously verified.

This study addresses this gap by exploring the hypothesis that SIRT3‐dependent mitophagic flux contributes to RIPC's renoprotective effects against renal IRI. Utilizing integrative in vivo and in vitro models of renal IRI, coupled with pharmacological inhibition of autophagy, SIRT3 gene knockout, rescue experiments and mitophagic flux verification assays, we sought to delineate the mechanistic cascade linking RIPC to renal salvage. Our findings establish a functional relationship between the SIRT3‐mitophagy axis and RIPC‐induced renal protection. Furthermore, these results suggest that SIRT3 may serve as a potential molecular target to optimize the efficacy of RIPC in mitigating renal IRI associated with PCI.

## 2 Materials and Methods

### 2.1 Animals and Models

This study has been reported in accordance with the ARRIVE guidelines (Animals in Research: Reporting In Vivo Experiments) [18]. Male C57BL/6 mice, 10 weeks old and weighing 20–25 g, were acquired from Beijing Sibeifu Biotechnology Co. Ltd. These mice were housed in a controlled environment with a 12 h light/dark cycle, at 24°C–26°C and 50% humidity. The study adhered to the Animal Ethics Committee of Baotou Medical College guidelines and conformed to national animal experiment regulations.

SIRT3 knockout mice, strain number KOCMP‐64384‐SIRT3‐B6N, were procured from Guangzhou Saiye Biotechnology Co. Ltd. Using CRISPR/Cas9, SIRT3 gene knockouts were generated. Mice were housed under conditions identical to those of the C57BL/6 mice, with free access to food and water. After acclimatization, breeding commenced by co‐housing a male and a female, resulting in offspring that underwent genotyping by PCR and agarose gel electrophoresis.

### 2.2 Study Group

C57BL/6 mice were randomly assigned to five groups, with 6–8 mice per group to ensure statistical power. Anaesthesia was induced with an intraperitoneal injection of pentobarbital sodium (70 mg/kg). Subsequent procedures were initiated once the tail clamping reflex was absent:

1. Sham group: Bilateral kidneys were exposed and manipulated without injury for 30 min, followed by suturing and 23 h of normal post‐operative care.
2. Ischaemia/reperfusion injury (IRI) group: Bilateral renal pedicles were clamped for 30 min to induce ischaemia, followed by reperfusion, suturing and normal post‐operative care for 23 h.
3. Remote ischaemic preconditioning (RIPC) group: Non‐invasive cuff‐induced limb ischaemia (four cycles of 5 min of ischaemia and 5 min of reperfusion) was performed, followed by bilateral renal ischaemia/reperfusion as in the IRI group.
4. RIPC + 3‐MA group: In addition to RIPC treatment, 3‐methyladenine (3‐MA; 30 mg/kg, 0.2 mL/20 g, prepared at 3 mg/mL) was administered intraperitoneally 2 h before the IRI procedure.
5. RIPC + SIRT3 KO group: 6–8 SIRT3 knockout (KO) mice were used for this group, which underwent RIPC before the IRI procedure; after IRI, mice received suturing and 23 h of normal post‐operative care.

### 2.3 Experimental Models of Renal IRI in Mice

Intraperitoneal injection of pentobarbital sodium (70 mg/kg) was used for anaesthesia. Once the tail clamping reflex vanished, an incision was made to expose the kidneys. During surgery, core body temperature was maintained at 36.5°C–37.5°C using a thermostatically controlled heating pad with rectal probe monitoring. For post‐operative analgesia, buprenorphine (0.05 mg/kg) was administered subcutaneously immediately before recovery from anaesthesia and again at 12‐h intervals for 24 h post‐surgery [2]. Ischaemia was induced by clamping the renal pedicle for 30 min, followed by reperfusion. The colour change of the kidney from bright red to dark purple signified successful ischaemia, and a return to pink indicated successful reperfusion [6].

### 2.4 RIPC Induction, Extraction and Preservation of RIPC Isolates

RIPC involved repeated cycles of non‐lethal ischaemia and reperfusion on a distal organ, here the right hind limb, using a non‐invasive cuff to occlude blood flow (four cycles of 5 min ischaemia/5 min reperfusion) 2 h before the renal IRI. Successful limb ischaemia was confirmed visually by the change in skin colour from pink to purple during the occlusion period, and successful reperfusion was confirmed by the return to pink colour upon cuff release. Blood was collected via cardiac puncture 1 h post‐RIPC, centrifuged at 1000 *g* for 20 min at 4°C to separate plasma, which was then aliquoted and stored at −80°C in heparinized Eppendorf tubes. This RIPC plasma intervention is a standard in vitro mimicry strategy for in vivo RIPC effects, which recapitulates the humoral/paracrine protective signals released by limb RIPC in vivo and enables the investigation of RIPC‐mediated cellular and molecular mechanisms in cultured HK‐2 cells [7, 8].

### 2.5 Autophagy Inhibitor Treatment Method

3‐Methyladenine (3‐MA), a PI3K inhibitor and early‐stage autophagy blocker, was injected intraperitoneally (0.2 mL/20 g, prepared at 3 mg/mL) 2 h before IRI induction. Chloroquine (CQ; Sigma‐Aldrich), a late‐stage autophagy inhibitor that blocks autophagosome‐lysosome fusion, was dissolved in sterile PBS to a stock concentration of 100 mM and used at a final concentration of 50 μM for in vitro cell experiments [19, 20].

### 2.6 Mitochondrial Isolation, Extraction and Identification

Mitochondria were isolated from renal tissues using a mitochondrial extraction kit (EX1321; Solarbio) according to the instructions. Briefly, 50–100 mg of renal cortex tissue was harvested, minced and homogenized in 1 mL of ice‐cold Buffer A using a small‐volume glass homogenizer (30–40 strokes). The homogenate was subjected to differential centrifugation as follows: 500 × *g* for 5 min at 4°C to pellet nuclei and unbroken cells; the supernatant was collected and centrifuged at 1000 × *g* for 10 min at 4°C; the resulting supernatant was then centrifuged at 10,000 × *g* for 20 min at 4°C to obtain the crude mitochondrial pellet. The pellet was resuspended in 200 μL of ice‐cold Buffer B and centrifuged again at 10,000 × *g* for 20 min at 4°C. The washed mitochondrial pellet was resuspended in 150 μL of Buffer C and incubated with gentle shaking for 20–40 min at 4°C. After a final centrifugation at 10,000 × *g* for 10 min at 4°C, the supernatant containing purified mitochondrial proteins was collected and aliquoted for subsequent analyses. All steps were performed at 4°C to maintain mitochondrial integrity [2].

### 2.7 JC‐1 Mitochondrial Membrane Potential Assay

Mitochondrial membrane potential was examined by JC‐1 (KeyGEN BioTECH) in live cells. HK‐2 cells were seeded in 6‐well plates and treated with corresponding interventions before JC‐1 staining. After HK‐2 cell line post‐incubation, cells were examined by fluorescence microscopy (Nikon LV150N). For quantitative analysis, 8–10 fields (200×) were randomly selected in each condition. The red to green JC‐1 fluorescence ratio was measured using Image J, with a higher ratio indicating intact mitochondrial membrane potential.

### 2.8 TUNEL Assay

Apoptotic cells were identified by TUNEL assay using an in situ cell death detection kit (KeyGEN BioTECH KGA7071) for tissue sections. For HK‐2, apoptosis was determined employing the Annexin V‐FITC apoptosis detection kit according to the supplier's instructions. Initially, tissue sections were deparaffinized and permeabilized with 0.1 M sodium citrate (pH 6.0, heated at 95°C for 15 min). The sections were then incubated with a TUNEL reaction mixture. Positive nuclear staining was detected by fluorescence microscopy, with quantification based on TUNEL‐positive cells per mm^2^. Ten to twenty fields (200×) were randomly selected from each tissue section for blind counting.

### 2.9 Immunofluorescence Staining for Parkin/LC3B Colocalization

Renal tissue sections or HK‐2 cells were used for immunofluorescence staining to detect the colocalization of Parkin and LC3B. For tissue sections: deparaffinization, rehydration and antigen retrieval were performed sequentially; for cultured HK‐2 cells: fixed with 4% paraformaldehyde for 15 min, permeabilized with 0.1% Triton X‐100 for 10 min, and blocked with 5% bovine serum albumin (BSA) for 1 h at room temperature. The samples were incubated with primary antibodies against Parkin and LC3B (1:200 dilution) at 4°C overnight, then incubated with fluorescence‐conjugated secondary antibodies (Alexa Fluor 488 for LC3B, Alexa Fluor 594 for Parkin, 1:500 dilution) for 1 h at room temperature in the dark. DAPI (1:1000) was used for nuclear counterstaining. Fluorescent images were captured under a laser scanning confocal microscope (Nikon A1). The colocalization efficiency of Parkin and LC3B was quantified using Image J software (Coloc 2 plugin), and the Pearson's correlation coefficient was calculated to evaluate the colocalization level. Eight to ten random fields (400×) were selected for each group for blind quantitative analysis.

### 2.10 Western Blot

Proteins were extracted from renal tissues, HK‐2 cells or isolated mitochondrial fractions using RIPA buffer with inhibitors. After BCA quantification, equal protein amounts (30 μg) were subjected to SDS‐PAGE, transferred to PVDF membranes and immunoblotted with antibodies against LC3B, p62, SIRT3, cleaved‐caspase‐3, caspase‐3 and β‐actin. Blots were visualized by ECL and quantified using ImageJ. Each group comprised at least three independent experiments.

### 2.11 qRT‐PCR

Total RNA was extracted from cells or tissues utilizing the TRIzol lysis solution, followed by reverse transcription into cDNA using a Fermentas reverse transcription kit. The synthesized cDNA was then amplified via qRT‐PCR on an ABI‐7300 qRT‐PCR System. Quantification of gene expression was performed using the 2^−ΔΔCt^ method.

For mouse tissue samples, the following primer sequences were used: SIRT3 (forward: 5′‐ACGGGCTTGAGAGAGCATCT‐3′; reverse: 5′‐TCGGGTTTCACAACGCCAGT‐3′; product size: 178 bp). For HK‐2 cell samples, the following primer sequences were used: GAPDH (forward: 5′‐TCAACGGCACAGTCAAGG‐3′; reverse: 5′‐TGAGCCCTTCCACGATG‐3′), Beclin1 (forward: 5′‐ATCCTGGACCGAGTGACCATTC‐3′; reverse: 5′‐TCTCCTGAGTTAGCCTCTTCCTCC‐3′), LC3 (forward: 5′‐GCCTGTCCTGGATAAGACCAA‐3′; reverse: 5′‐ACCATGCTGTGCTGGTTCAC‐3′). All primers were synthesized by Sangon Biotech (Shanghai, China).

### 2.12 Kidney Histological Examination

Kidney tissues were fixed with 4% paraformaldehyde, embedded in paraffin, sectioned at 4 μm and stained with H&E. Tubular damage was assessed blindly by two pathologists based on percentage of damaged tubules: 0 (no damage), 1 (< 25%), 2 (25%–50%), 3 (51%–75%), 4 (> 75%).

### 2.13 Biochemical Analyses

Blood samples, obtained via open‐heart collection at room temperature, were centrifuged to separate serum. Renal function parameters, including blood urea nitrogen (BUN) and serum creatinine (Ser) levels, were quantitatively assessed using an Abbott C16000 automatic blood biochemical analyzer.

### 2.14 Transmission Electron Microscope

For transmission electron microscopy (TEM) analysis, renal tissues were fixed in glutaraldehyde, followed by post‐fixation in osmium tetroxide. Subsequently, the samples were dehydrated in a graded ethanol series and embedded in epoxy resin. Ultrathin sections were prepared, stained with uranyl acetate and lead citrate. The samples were analysed using TEM to assess ultrastructural alterations and identify autophagic vacuoles. The number of autophagosomes and autolysosomes in renal tubular epithelial cells was counted in 30 random fields per group for quantitative analysis.

### 2.15 Cell Culture and Transfection

HK‐2 cells were cultured in CM‐0109 medium supplemented with 10% foetal bovine serum and 1% penicillin–streptomycin, maintained at 37°C in a humidified atmosphere containing 5% CO_2_. For gene silencing, SIRT3‐siRNA was transfected into HK‐2 cells using Lipofectamine 8000 following the manufacturer's instructions. For SIRT3 overexpression experiments, HK‐2 cells were transfected with the SIRT3 overexpression plasmid (GenePharma, Shanghai) or the empty vector plasmid using Lipofectamine 8000.

Before transfection, cells were seeded in culture dishes to reach 70%–80% confluence. 8000 DNA‐Lipofectamine complexes were prepared in serum‐free medium and then added to the cells. After 4–6 h of incubation, the medium was replaced with complete growth medium. Cells were cultured for 24–48 h post‐transfection before subsequent experimental analyses. The efficiency of SIRT3 knockdown and overexpression was verified by Western blot and qRT‐PCR [2].

### 2.16 H/R‐Induced Cellular Injury Model

A hypoxia/reoxygenation‐induced cellular injury model was established via physical methods. HK‐2 cells in good condition were seeded in 6‐well or 96‐well plates and pre‐treated with RIPC plasma or CQ as required, then cultured under hypoxic conditions (1% O_2_, 94% N_2_ and 5% CO_2_) at 37°C for 15 h, followed by reoxygenation in a normoxic incubator (5% CO_2_ and 95% air) at 37°C for 6 h. A RIPC group was set up in which HK‐2 cells were treated with RIPC plasma alone without H/R induction to verify the specificity of RIPC effects.

### 2.17 Statistical Analysis

Quantitative data were expressed as means ± standard deviation (SD). Statistical analysis was performed utilizing GraphPad Prism software. For multiple group comparisons among wild‐type (WT) mice (Sham, IRI, RIPC + IRI, 3‐MA + RIPC + IRI), one‐way analysis of variance (ANOVA) followed by Tukey's post hoc test was used for pairwise comparisons. For direct comparison between WT and SIRT3 knockout (KO) mice under the identical RIPC + IRI treatment condition, an unpaired two‐tailed Student's *t*‐test was employed. For in vitro cell experiments, one‐way ANOVA with Tukey's post hoc test was used for multiple group comparisons, and an unpaired two‐tailed Student's *t*‐test for two‐group comparisons. A *p*‐value < 0.05 was considered statistically significant, marked as *p < 0.05 and **p < 0.01.

## 3 Results

### 3.1 RIPC Has Significant Kidney Protective Effects Through Cell Apoptosis Inhibition

The mice were subjected to bilateral renal ischaemia for 30 min, followed by reperfusion for 24 h to establish a mouse renal ischaemia–reperfusion model (Figure 1A). A total of 6–8 male mice were included in each experimental group (Sham, IRI, RIPC + IRI, 3‐MA + RIPC + IRI, SIRT3 KO + RIPC + IRI) to ensure statistical power. A small number of mice (*n* = 2 total across all groups) died unexpectedly during the experiment; these animals were excluded, and only data from mice that completed the experiment (without severe adverse events) were included in the final analysis. All quantitative data are presented as mean ± standard deviation (SD), with statistical significance set at *p* < 0.05 (detailed statistical methods in Section 2). In the analysis of renal function, renal IRI caused severe kidney damage, and blood urea nitrogen (BUN) and serum creatinine (Scr) levels increased to 26.47 ± 10.31 mmol/L and 151.90 ± 41.99 μmol/L, respectively (Figure 1B,C, IRI vs. Sham, *p* < 0.001). RIPC pretreatment significantly decreased serum BUN and Scr levels to 14.69 ± 7.74 mmol/L and 100.90 ± 26.51 μmol/L (Figure 1B,C, RIPC + IRI vs. IRI, *p* < 0.05). In histological analysis with haematoxylin–eosin (HE) staining, renal IRI‐induced massive swelling and necrosis of renal tubules, and RIPC pretreatment markedly alleviated this tubular injury (Figure 1D,E). Two independent professional pathologists performed semi‐quantitative scoring in a blind manner; the tubular damage scores in the IRI and RIPC + IRI groups were 2.67 ± 0.82 and 1.83 ± 0.41 (*p* < 0.05). TUNEL staining revealed that renal RIPC significantly attenuated tubular epithelial cell apoptosis in renal IRI (Figure 1F). Quantitatively, RIPC reduced the number of TUNEL‐positive cells per square millimetre of renal tissue from 27.00 ± 14.09 to 10.92 ± 2.32 (*p* < 0.05) (Figure 1F). To further verify the anti‐apoptotic effect of RIPC, we detected the expression of cleaved‐caspase‐3, a key apoptosis‐specific protein, in renal tubular epithelial cells of each group. The results showed that IRI significantly upregulated the expression of cleaved‐caspase‐3, and RIPC pretreatment effectively reversed this upregulation (Figure 1H–J, *p* < 0.05). Together, these results confirmed the significant renoprotective effect of RIPC in our experimental setting. The inhibitory effect of RIPC on renal tubular epithelial cell apoptosis may be a major mechanism underlying its improvement of renal function after IRI.

**FIGURE 1:**
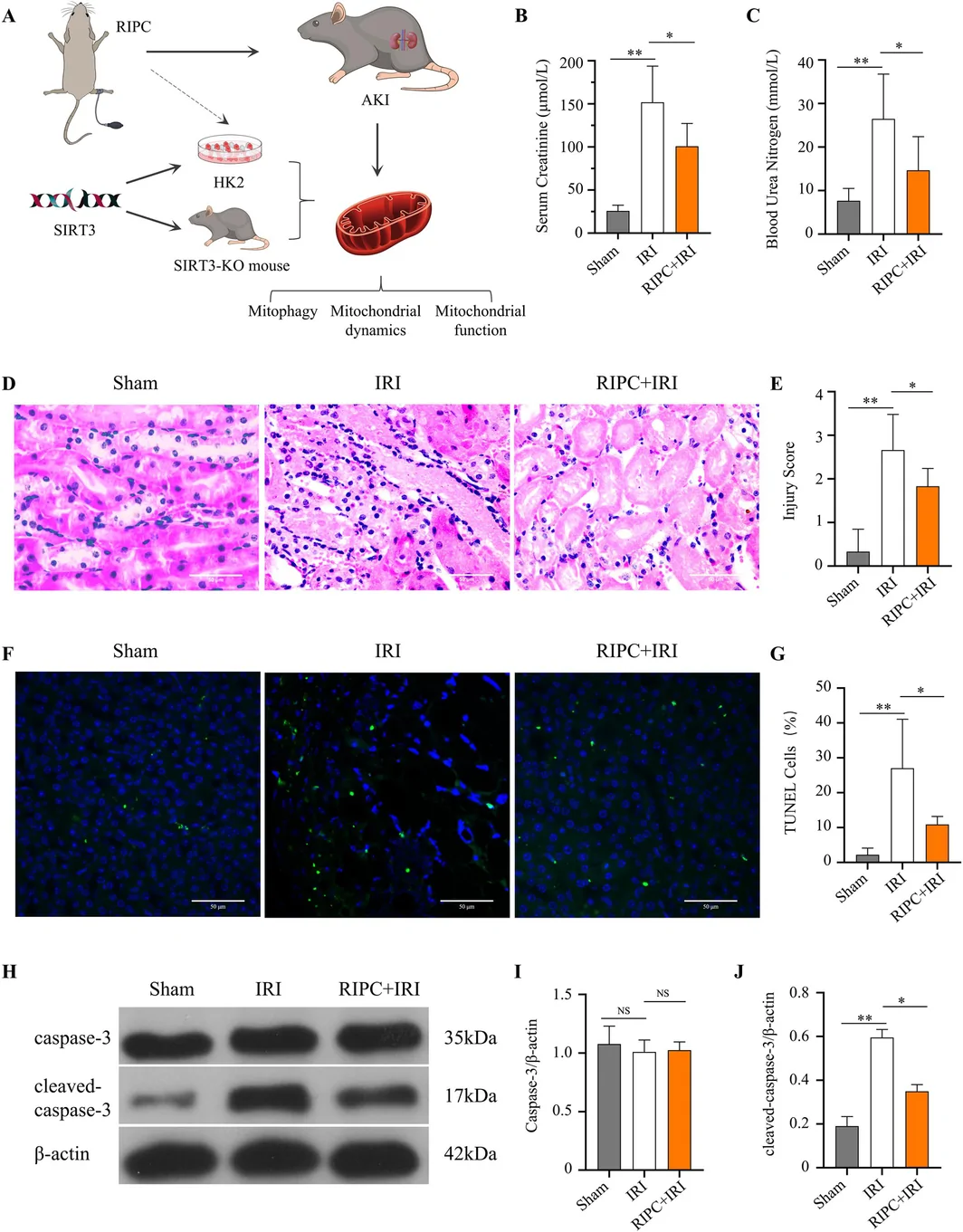
RIPC protects the kidney through cell apoptosis inhibition. (A) A simplified schematic of this study. (B) Levels of Scr in serum of mice in each group; (C) Levels of BUN in serum of mice in each group. (D, E) Histopathological examination (H&E staining) results of mice in each group (400×), Scale bar: 50 μm. Blind semi‐quantitative renal injury scores were evaluated by two independent pathologists. (F, G) Apoptosis level of renal tubule cells in each group (TUNEL staining, 400×), Scale bar: 50 μm. Quantitative analysis of TUNEL‐positive cells per mm^2^ of renal tissue. (H) Representative Western blot bands showing the expression of caspase‐3/cleaved‐caspase‐3 protein in mitochondria of renal tubular epithelial cells of mice in each group; β‐actin was used as a loading control. (I, J) Quantitative analysis of caspase‐3 (I) and cleaved caspase‐3 (J) protein expression levels. All data are expressed as mean ± SD. *p < 0.05, **p < 0.01; NS, not significant.

### 3.2 RIPC Could Activate Mitophagy in Proximal Tubules to Reduce Kidney Injury

Firstly, JC‐1 staining was used to detect the mitochondrial membrane potential (MMP) and assess mitochondrial functional state in renal tubular epithelial cells. Under a fluorescence microscope, it showed red fluorescence when the mitochondrial membrane potential was high, indicating that the cell state was in good condition. Green fluorescence at low mitochondrial membrane potential indicates impaired mitochondrial function. The red fluorescence/green fluorescence ratio was used to represent the mitochondrial membrane potential level. Renal IRI significantly increased the number of dysfunctional mitochondria and reduced the MMP ratio, while RIPC pretreatment markedly elevated the MMP ratio and increased the number of functionally intact mitochondria (Figure 2A,B, Sham vs. IRI, RIPC + IRI vs. IRI, *p* < 0.05), suggesting a direct protective effect of RIPC on renal mitochondrial function. However, intraperitoneal injection of 3‐MA 2 h before RIPC to inhibit early autophagy completely abrogated the renoprotective effect of RIPC, which was manifested as a significant decrease in the MMP ratio (Figure 2B, 3‐MA + RIPC + IRI vs. RIPC + IRI, *p* < 0.05). Consistently, the renoprotective effect of RIPC was also blocked by 3‐MA pretreatment, as the serum BUN and Scr levels in the 3‐MA + RIPC + IRI group were elevated to 29.34 ± 10.59 mmol/L and 146.50 ± 80.35 μmol/L, respectively (Figure 2C,D, 3‐MA + RIPC + IRI vs. RIPC + IRI, *p* < 0.05). These results preliminarily demonstrate that mitophagy plays a critical role in mediating the renoprotective effect of RIPC against renal IRI.

**FIGURE 2:**
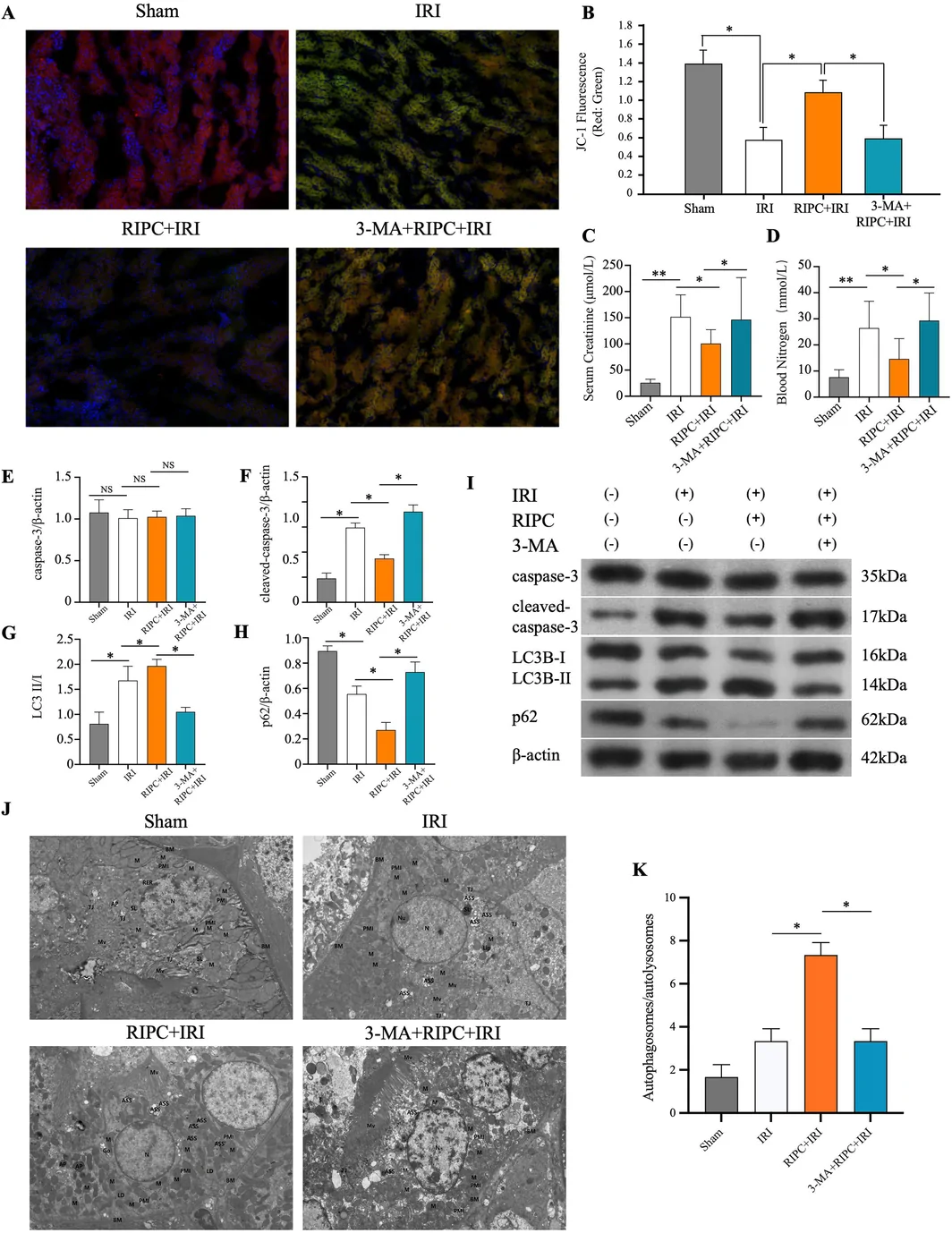
RIPC activates mitophagy in renal proximal tubular epithelial cells against IRI. (A, B) Mitochondrial membrane potential level of JC‐1 (400×). (C, D) Levels of Scr and BUN in the serum of mice in each group. (E–I) Expression of caspase‐3/cleaved‐caspase‐3, LC3 II/I, p62 protein in mitochondria of renal tubular epithelial cells of mice in each group; β‐actin was used as a loading control. (J) Autophagosome and autolysosome were observed under transmission electron microscope; AP, autophagic corpuscle; ASS, autolysosome; BM, basement membrane; M, mitochondrion; Mv, microvilli; N, nucleus; RER, rough endoplasmic reticulum; SL, secondary lysosome; Ruler = 5 μm. (K) Quantitative analysis of autophagosomes/autolysosomes per unit cell area (*n* = 30 random fields, blind counting). All data are expressed as mean ± SD. *p < 0.05, **p < 0.01; NS, not significant.

To further analyse the renal protective mechanism of RIPC, Western blot was used to detect the expression levels of autophagy‐specific proteins LC3 II/I and p62 and apoptosis‐specific proteins caspase‐3 and cleaved‐caspase‐3 in mitochondria of mouse renal tubular epithelial cells (Figure 2I), with the detection of mitochondrial proteins specifically reflecting the level of mitochondrial autophagy. The results showed that compared with the IRI group, the RIPC + IRI group exhibited a significant increase in the LC3 II/I protein ratio, a marked decrease in p62 protein expression, and a significant reduction in cleaved‐caspase‐3 expression (Figure 2F–H, all *p* < 0.05). In contrast, compared with the RIPC + IRI group, the 3‐MA + RIPC + IRI group showed a significant decrease in the LC3 II/I ratio, a notable increase in p62 protein expression, and a marked upregulation of cleaved‐caspase‐3 expression (Figure 2F–H, all *p* < 0.05). These results confirm that RIPC can activate mitochondrial autophagy and inhibit apoptosis in renal tubular epithelial cells to exert kidney protective effects.

Transmission electron microscopy (TEM) was used to observe the ultrastructural changes of renal tubular epithelial cells and the status of mitochondrial autophagy in each group. Autophagosomes were defined as double‐membrane vesicles containing damaged mitochondria or cytoplasmic components, and autolysosomes as single‐membrane vesicles with degraded internal contents (Figure 2J). The Sham group showed mild mitochondrial swelling in proximal tubular cells with a small number of autophagosomes and abundant reabsorption vacuoles in the apical cytoplasm; the IRI group exhibited moderate mitochondrial swelling with a slight increase in autophagosome number; the RIPC + IRI group presented with minimal mitochondrial swelling and a marked increase in the number of autophagosomes/autolysosomes, indicating enhanced mitochondrial mitophagic flux; the 3‐MA + RIPC + IRI group showed moderate mitochondrial swelling and only a few autophagosomes/autolysosomes, suggesting inhibited mitochondrial autophagy. Quantitative analysis of the number of autophagosomes/autolysosomes per unit cell area in each group was shown in Figure 2K (*n* = 30 random fields per group for blind counting, all *p* < 0.05), which was consistent with the ultrastructural observations and Western blot results of mitochondrial mitophagy‐related proteins.

### 3.3 In Vitro RIPC Attenuates H/R‐Induced Injury in Renal Tubular Epithelial Cells

To further confirm the renoprotective effect of RIPC on renal epithelial cell injury, a hypoxia/reoxygenation (H/R) injury model was established using human renal tubular epithelial HK‐2 cells. HK‐2 cells were pre‐treated with rat RIPC plasma isolate to investigate the protective effect of RIPC against H/R‐induced cellular damage. Under an inverted microscope, the H/R group showed significant morphological changes compared with the control group: cells exhibited swelling and degeneration, with blurred nuclear boundaries and extensive cell death. In contrast, the RIPC + H/R group maintained normal cellular morphology, clear nuclear boundaries and markedly reduced cell death (Figure 3A). Flow cytometry (FACS) analysis of cellular apoptosis was consistent with the morphological observations: compared with the control group, the H/R group had a significant increase in apoptotic rate (*p* < 0.01), while the RIPC + H/R group showed a marked decrease in apoptotic rate compared with the H/R group (*p* < 0.01) (Figure 3B,C). These results indicated that the RIPC plasma isolate exerts a protective effect against H/R‐induced injury in HK‐2 cells.

**FIGURE 3:**
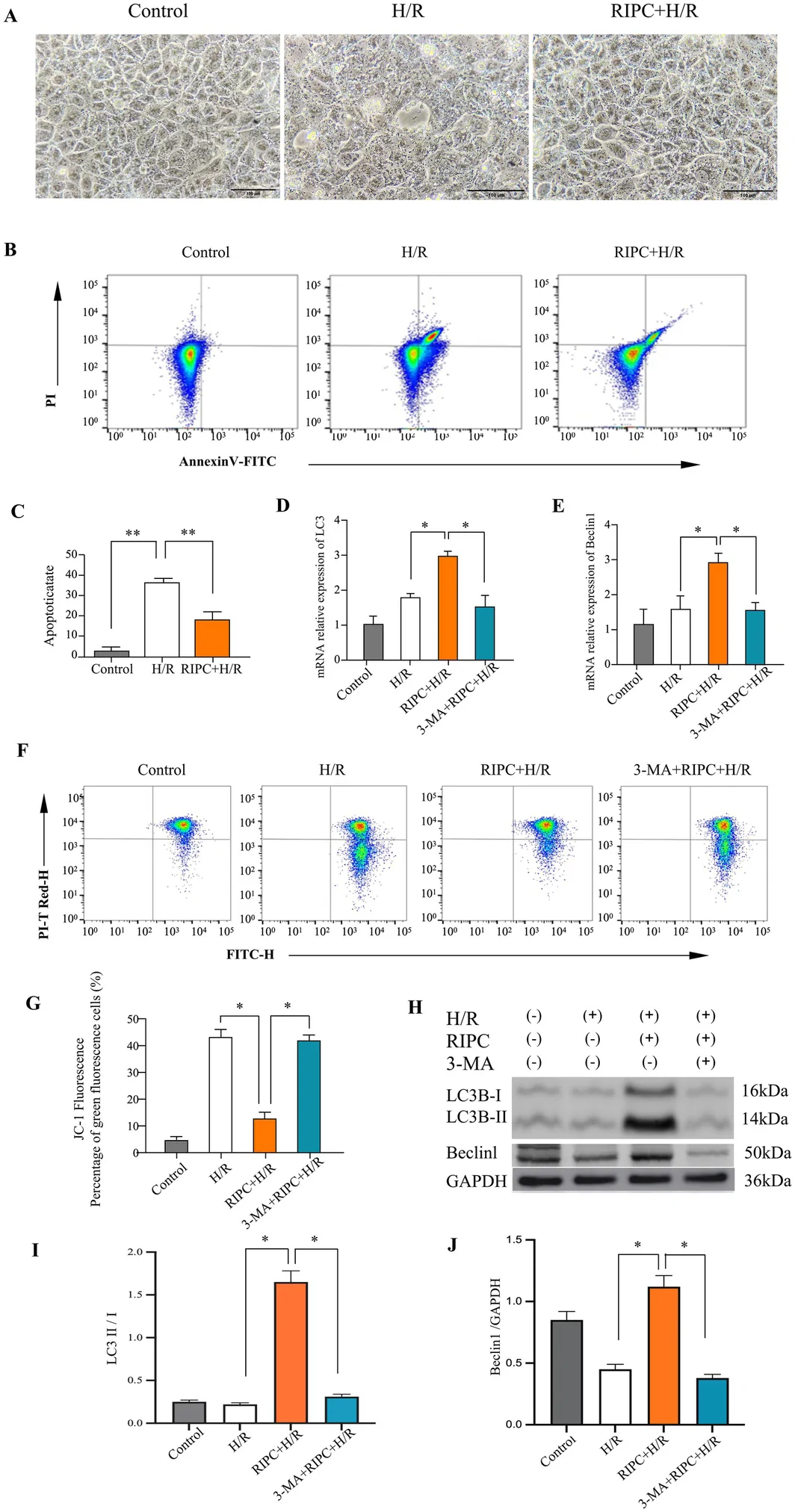
RIPC isolate protects HK‐2 cells against H/R injury via SIRT3‐mediated mitochondrial mitophagy. (A) Morphological changes of HK‐2 cells in each group under an inverted microscope (400×), Scale bar: 100 μm. (B, C) Apoptotic rate of HK‐2 cells in each group detected by flow cytometry (FACS). (D, E) mRNA expression levels of LC3 and Beclin1 in mitochondrial fractions of HK‐2 cells in each group were detected by qRT‐PCR. (F, G) Mitochondrial membrane potential (MMP) of HK‐2 cells in each group was detected by JC‐1 probe and FACS analysis (*X*‐axis: FITC‐H [green, JC‐1 monomers]; *Y*‐axis: PI‐T Red‐H [red, JC‐1 aggregates]). Quantitative analysis of the percentage of green fluorescence cells (indicating mitochondrial depolarization). (H) Representative Western blot bands of mitochondrial proteins showing the expression of LC3B and Beclin1 in HK‐2 cells of each group; GAPDH was used as a loading control. (I, J) Quantitative analysis of LC3II/I ratio (I) and Beclin1/GAPDH relative protein expression (J). All data are expressed as mean ± SD. *p < 0.05, **p < 0.01; NS, not significant.

To elucidate the mechanistic basis of this protection, we first examined mitophagy‐related gene expression. Quantitative RT‐PCR analysis revealed that compared with the H/R group, the RIPC + H/R group had significantly upregulated mRNA expression of LC3 and Beclin1 (*p* < 0.05). In contrast, the 3‐MA + RIPC + H/R group exhibited significantly reduced expression of these genes compared with the RIPC + H/R group (*p* < 0.05) (Figure 3D,E).

Mitochondrial membrane potential (MMP) is a key indicator for evaluating mitochondrial functional status; thus, we used the cell‐permeable fluorescent probe JC‐1 to detect changes in MMP in HK‐2 cells. In functionally intact mitochondria, JC‐1 forms red fluorescent aggregates, while in damaged mitochondria, it exists as green fluorescent monomers. The percentage of cells with green fluorescence was analysed by flow cytometry to indicate mitochondrial depolarization. The results showed that compared with the H/R group, the RIPC + H/R group exhibited a significantly lower percentage of green fluorescent cells (*p* < 0.05), indicating preserved MMP and improved mitochondrial function. In contrast, the 3‐MA + RIPC + H/R group showed a marked increase in the percentage of green fluorescent cells compared with the RIPC + H/R group (*p* < 0.05) (Figure 3F,G), suggesting that mitophagy inhibition abolished the mitochondrial protective effect of RIPC.

Finally, we validated these findings at the protein level by Western blotting of mitochondrial fractions. Consistent with the transcriptional data, the RIPC + H/R group showed significantly increased protein expression of LC3 II/I and Beclin1 compared with both the H/R group and the 3‐MA + RIPC + H/R group (Figure 3H–J, *p* < 0.05). Collectively, these results demonstrate that the RIPC plasma isolate protects HK‐2 cells from H/R‐induced damage by activating mitochondrial mitophagy in renal tubular epithelial cells.

### 3.4 In Vitro SIRT3 Promotes Mitophagy in HK‐2 Cells During RIPC Treatment

The protective role of mitophagy in acute kidney injury (AKI) has been well established, yet the specific molecular regulators mediating mitophagic activation in renal tubular epithelial cells remain incompletely elucidated. Sirtuin 3 (SIRT3), an NAD^+^‐dependent mitochondrial deacetylase, is a key modulator of mitochondrial homeostasis that positively regulates multiple mitochondrial processes, including mitochondrial biogenesis, oxidative stress resistance, and energy metabolism [21]. Given its core role in regulating mitochondrial mitophagy and close association with renal IRI protection, SIRT3 was identified as the key molecular target in this study to investigate the mechanistic link between RIPC, mitophagic activation and renal epithelial cell protection against H/R injury.

To determine whether SIRT3 is required for RIPC‐induced protection and mitochondrial mitophagy, we performed SIRT3 knockdown in HK‐2 cells followed by RIPC pretreatment and H/R injury. Morphological observation under an inverted microscope revealed that the si‐SIRT3 + RIPC + H/R group exhibited marked cellular swelling, degeneration, and increased cell death compared with the RIPC + H/R group shown in Figure 3A (Figure 4A). Western blot analysis confirmed that SIRT3 protein expression was significantly reduced in the si‐SIRT3 + RIPC + H/R group compared with the RIPC + H/R group (Figure 4B), verifying successful SIRT3 knockdown efficiency. Quantitative analysis of SIRT3 expression further validated these results (*p* < 0.05, Figure 4C).

**FIGURE 4:**
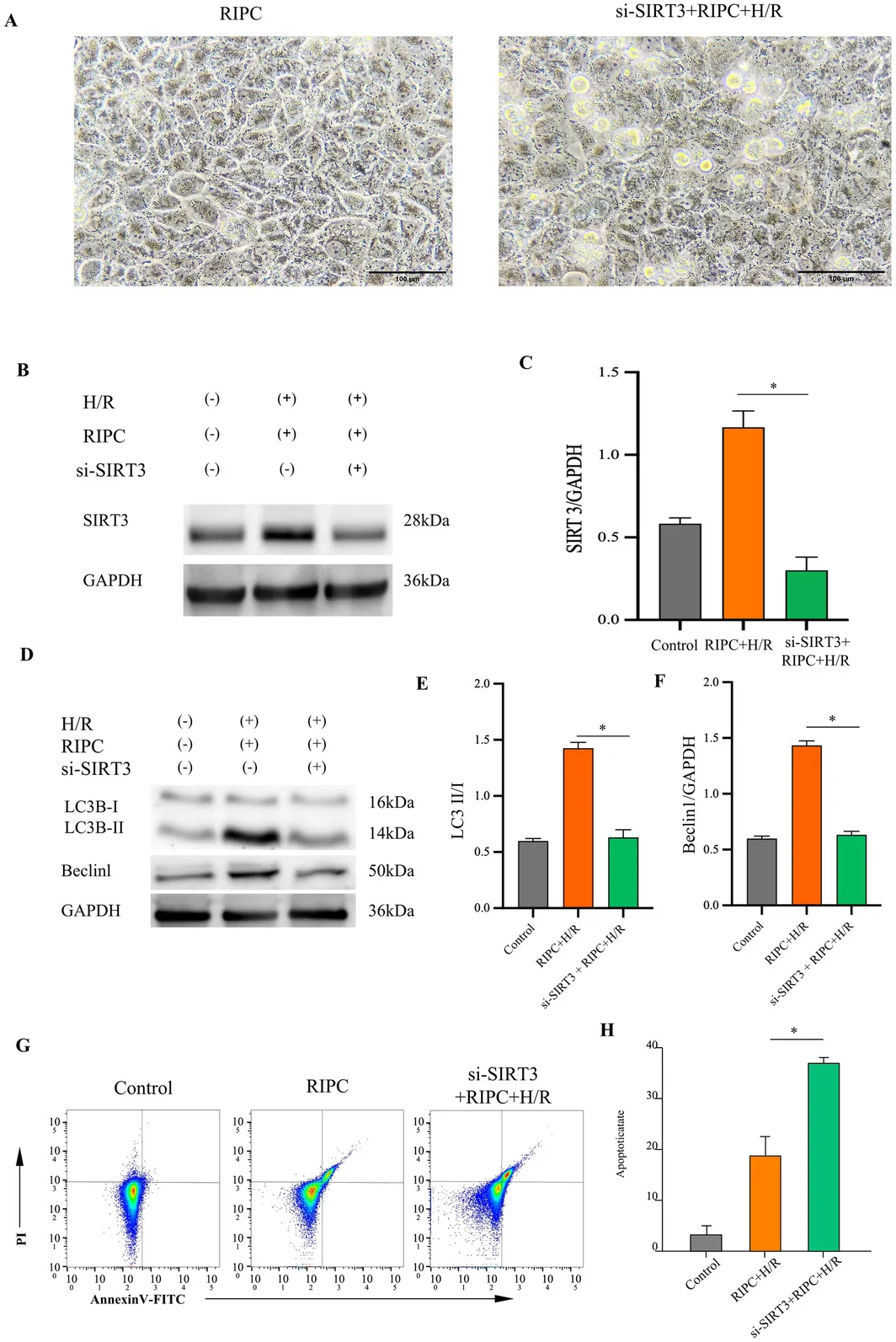
SIRT3 is an essential mediator of RIPC‐induced mitochondrial mitophagy in HK‐2 cells against H/R injury in vitro. (A) Representative image of the si‐SIRT3+RIPC+H/R group (400×, 100 µm); RICP+H/R is shown in Figure 3A. (B) Representative Western blot bands of SIRT3 and GAPDH in HK‐2 cells (GAPDH as loading control). (C) Quantitative analysis of SIRT3/GAPDH relative protein expression. (D) Representative Western blot bands of mitophagy‐related proteins Beclin1, LC3B and GAPDH in HK‐2 cells (GAPDH as loading control). (E, F) Quantitative analysis of LC3II/I ratio (E) and Beclin1/GAPDH relative protein expression (F). (G, H) Flow cytometry analysis of apoptotic levels of HK‐2 cells in each group (G) and quantitative analysis of apoptotic rate (H). All data are expressed as mean ± SD. *p < 0.05, **p < 0.01; NS, not significant.

We then examined the effect of SIRT3 knockdown on mitophagy‐related proteins. Western blot analysis of mitochondrial fractions showed that the levels of LC3 II/I and Beclin1 were markedly decreased in the si‐SIRT3 + RIPC + H/R group compared with the RIPC + H/R group (Figure 4D). Quantitative analysis confirmed significant reductions in both LC3 II/I ratio (*p* < 0.05) and Beclin1 expression (*p* < 0.05) following SIRT3 knockdown (Figure 4E,F).

To assess the functional consequences of SIRT3 knockdown on cell injury, we measured apoptosis using flow cytometry. The results showed that SIRT3 knockdown significantly increased the apoptotic rate compared with the RIPC + H/R group (*p* < 0.05, Figure 4G,H), indicating exacerbated cellular injury.

Collectively, these results demonstrate that SIRT3 knockdown exacerbates H/R‐induced injury and apoptosis in HK‐2 cells and abolishes RIPC‐induced activation of mitochondrial mitophagy. Thus, SIRT3 is an essential mediator of the renoprotective and mitophagy‐promoting effects of RIPC in renal tubular epithelial cells.

### 3.5 The Renoprotective Effects of RIPC Are Abrogated in SIRT3KO Mice

SIRT3 global knockout (SIRT3 KO) mice were generated using CRISPR/Cas9 gene editing (Figure 5A). Genomic DNA was extracted from mouse tail tissue, and PCR amplification combined with agarose gel electrophoresis was performed to identify the genotype of offspring mice (Figure 5B,C). To confirm SIRT3 knockout efficiency at the transcriptional level, real‐time PCR was performed to detect SIRT3 mRNA expressions in renal tubular epithelial mitochondria. Compared with wild‐type mice, the SIRT3 KO group showed no obvious amplification signal, as demonstrated by the PCR amplification curves (Figure 5F) and quantitative analysis (Figure 5G), confirming successful and complete SIRT3 gene knockout.

**FIGURE 5:**
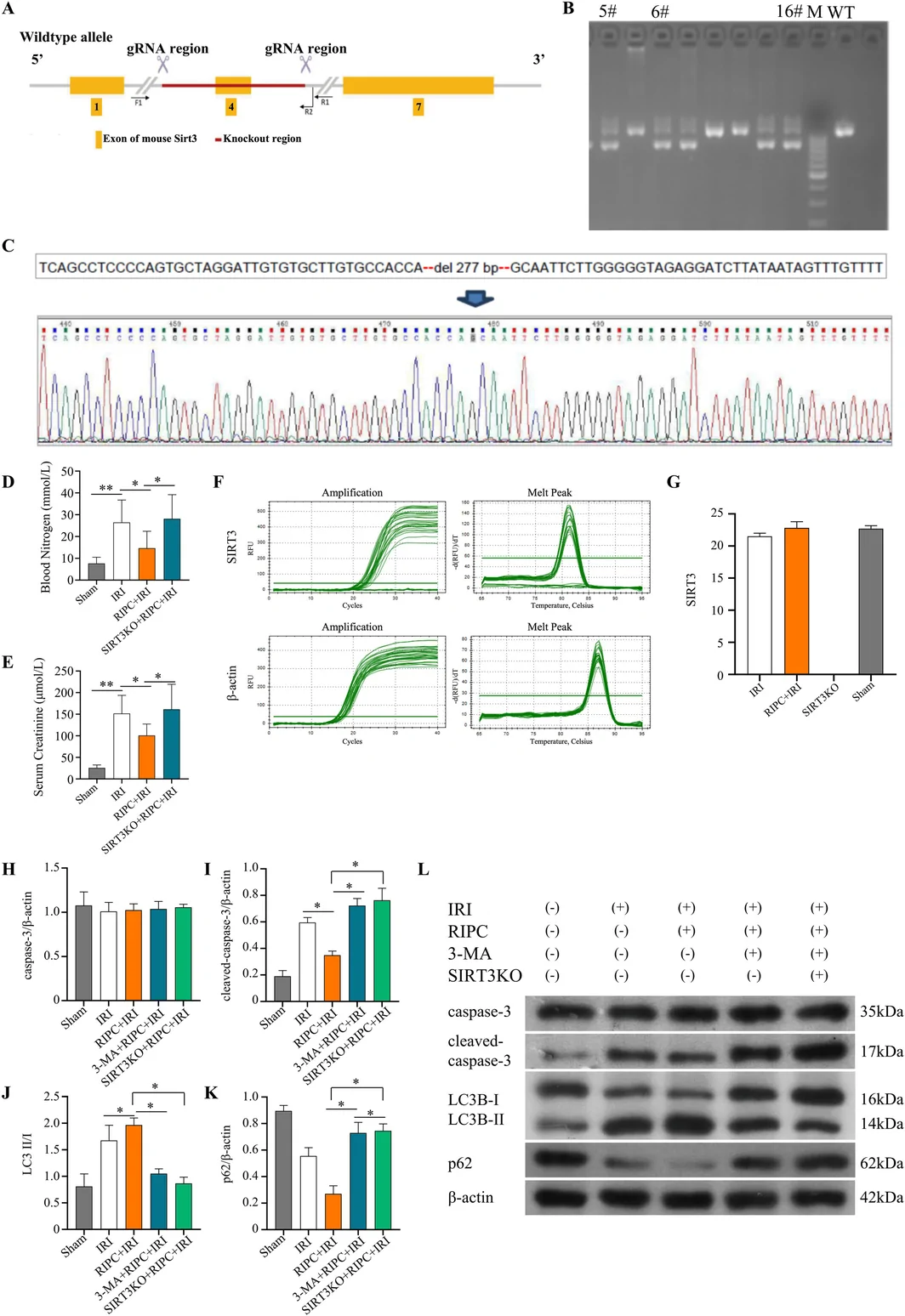
SIRT3 Knockout abolishes RIPC‐induced renoprotection against renal ischaemia–reperfusion injury. (A) Schematic diagram of SIRT3 gene knockout using the CRISPR/Cas9 system. (B, C) Genotype identification of offspring mice by PCR amplification and agarose gel electrophoresis. (D, E) Serum BUN and Scr level in each group. (F, G) SIRT3 mRNA expression in renal tubular epithelial mitochondria detected by real‐time PCR. (H–L) Expression of caspase‐3, cleaved‐caspase‐3, LC3 II/I and p62 in mitochondrial proteins of mouse renal tissue; β‐actin was used as a loading control. All data are expressed as mean ± SD. *p < 0.05, **p < 0.01; NS, not significant.

To determine the effect of SIRT3 deficiency on RIPC‐mediated renoprotection, renal function indicators including serum BUN and Scr were measured. Compared with the RIPC + IRI group, the RIPC + IRI + SIRT3 KO group exhibited significantly elevated serum BUN (28.19 ± 11.04 mmol/L) and Scr (161.60 ± 57.88 μmol/L) (Figure 5D,E, *p* < 0.05), indicating that SIRT3 knockout abolished the renal protective effect of RIPC.

To clarify the regulatory relationship between SIRT3 and mitochondrial mitophagy, the expression of mitophagy‐related proteins was detected in mitochondrial fractions. Compared with the RIPC + IRI group, the SIRT3 KO + RIPC + IRI group showed a significant increase in cleaved‐caspase‐3 expression (Figure 5H,I, *p* < 0.05), indicating exacerbated apoptosis following SIRT3 knockout. Regarding mitophagy‐related proteins, the SIRT3 KO + RIPC + IRI group exhibited a significant decrease in the LC3 II/I ratio (Figure 5J, *p* < 0.05) and a marked increase in p62 expression (Figure 5K, *p* < 0.05) compared with the RIPC + IRI group. Representative Western blot bands for cleaved‐caspase‐3, caspase‐3, LC3 II/I and p62 are shown in Figure 5L.

These results demonstrated that SIRT3 is essential for RIPC‐induced mitochondrial mitophagy and renoprotection in vivo.

### 3.6 RIPC Induces Complete Mitochondrial Mitophagy With Involvement of Parkin and SIRT3 to Exert Renoprotection

To further characterize the specificity, integrity of mitophagic flux and causal molecular mechanism of RIPC‐induced renoprotection, we performed a series of in vitro validation assays and explored the molecular mechanism in vivo. To exclude non‐specific effects of RIPC plasma isolate, we treated HK‐2 cells with RIPC in the absence of H/R injury; compared with the control group, RIPC treatment did not induce obvious morphological damage or apoptosis, and Western blot analysis of mitochondrial fractions showed no significant differences in the expression of SIRT3, LC3 II/I or p62 (*p* > 0.05, Figure 6A–D). These results confirmed that RIPC alone is not cytotoxic and does not affect basal mitochondrial mitophagy, supporting the specificity of RIPC‐induced protection during H/R injury. To verify whether RIPC enhances complete mitophagic flux rather than causing autophagic blockage, we used chloroquine (CQ), an inhibitor of late‐stage autophagosome‐lysosome fusion; compared with the RIPC + H/R group, the CQ + RIPC + H/R group exhibited a further significant increase in the LC3 II/I ratio (10.65 ± 1.21 vs. 3.62 ± 0.52, *p* < 0.05, Figure 6A,C) and marked accumulation of p62 protein in mitochondrial fractions (2.36 ± 0.16 vs. 0.90 ± 0.06, *p* < 0.05, Figure 6A,D). Together with our earlier 3‐MA results, these data confirmed that RIPC induces intact, functional mitophagic flux in renal tubular epithelial cells.

**FIGURE 6:**
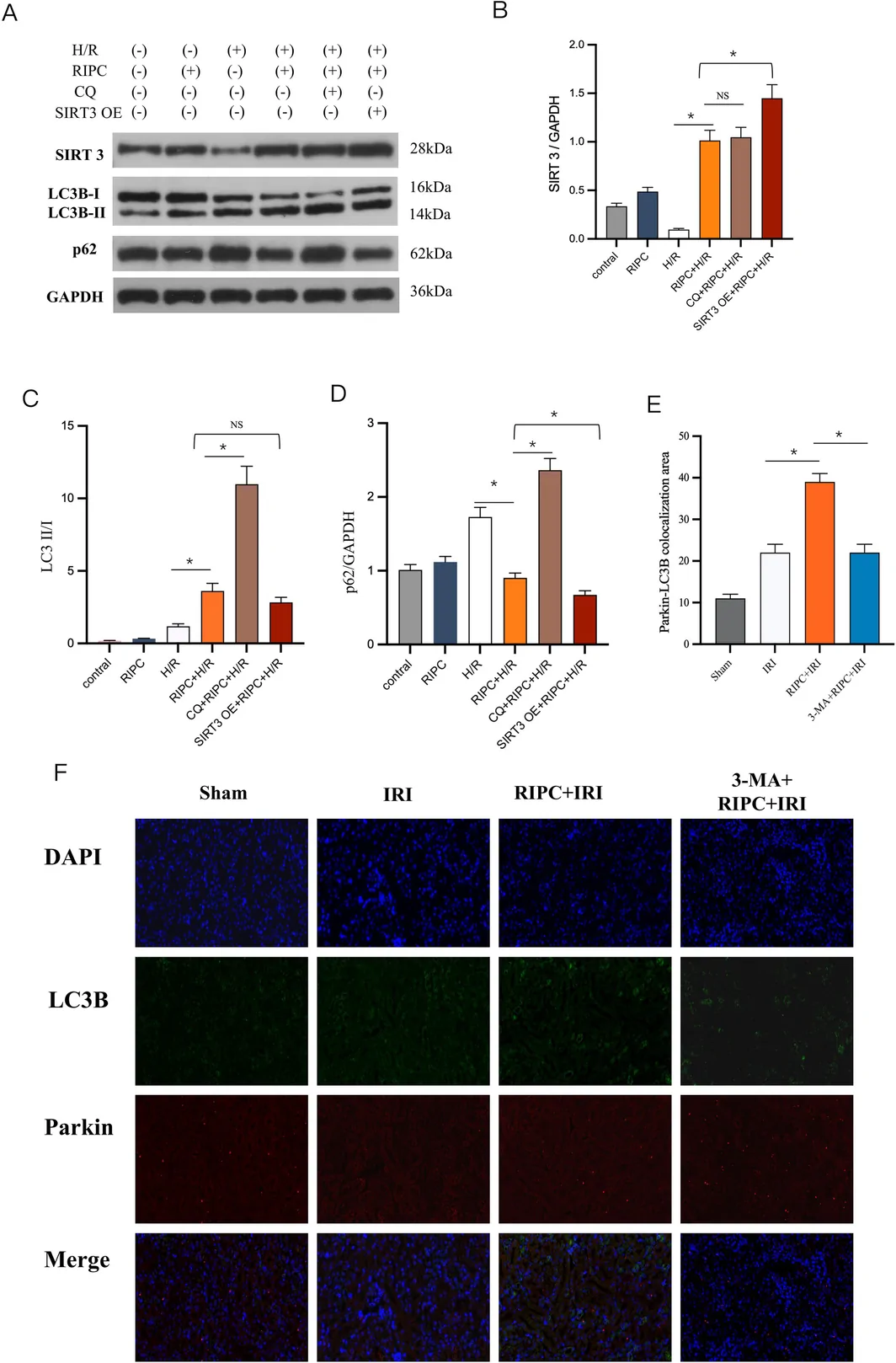
RIPC induces complete mitochondrial mitophagy with the involvement of SIRT3. (A) Representative Western blot bands of SIRT3, LC3B, p62 and GAPDH in HK‐2 cells of Control, RIPC, H/R, RIPC + H/R, CQ + RIPC + H/R and SIRT3 OE + RIPC + H/R groups (GAPDH as loading control). (B–D) Quantitative analysis of SIRT3/GAPDH relative expression (B), LC3II/I ratio (C) and p62/GAPDH relative expression (D) in HK‐2 cells. (E) Quantitative analysis of LC3B‐Parkin colocalization in mouse renal tissue. (F) Representative immunofluorescence images of LC3B (green) and Parkin (red) colocalization in mouse renal tissue (DAPI for nuclear staining, blue); scale bar consistent with experimental setting. All data are expressed as mean ± SD. *p < 0.05, **p < 0.01; NS, not significant.

To further confirm the causal regulatory role of SIRT3, we performed a SIRT3 overexpression (OE) experiment in HK‐2 cells. Compared with the RIPC + H/R group, SIRT3 OE significantly increased SIRT3 protein expression (1.45 ± 0.14 vs. 1.01 ± 0.10, *p* < 0.05), while maintaining the LC3 II/I ratio (2.83 ± 0.35 vs. 3.62 ± 0.52, *p* > 0.05) and p62 expression (0.67 ± 0.06 vs. 0.90 ± 0.06, *p* > 0.05) at levels comparable to RIPC + H/R group (Figure 6A–D). These gain‐of‐function data, together with our loss‐of‐function evidence from SIRT3 knockdown and knockout experiments, demonstrate that SIRT3 is essential for RIPC‐induced mitophagy and renoprotection.

Finally, to identify the key molecular mediator involved in RIPC‐induced mitochondrial mitophagy, we examined the colocalization of Parkin and LC3B in renal tubular epithelial cell mitochondria of mouse kidney tissues. The results showed that IRI significantly reduced the colocalization level of Parkin and LC3B in renal tissue mitochondria, whereas RIPC pretreatment markedly enhanced the colocalization of Parkin and LC3B. Moreover, the colocalization of Parkin and LC3B was significantly inhibited in the 3‐MA + RIPC + IRI group compared with the RIPC + IRI group (*p* < 0.05, Figure 6E,F), indicating that Parkin‐mediated mitophagosome formation in mitochondria is a critical process in RIPC‐induced mitophagic activation. Collectively, our findings demonstrate that RIPC specifically induces complete mitochondrial mitophagy in renal tubular epithelial cells with the synergistic involvement of Parkin and SIRT3, thereby protecting against renal ischaemia–reperfusion injury.

## 4 Discussion

Renal ischaemia–reperfusion injury (IRI) is a major contributor to PCI‐AKI, and exploring non‐invasive protective strategies with clear molecular mechanisms is of great clinical urgency. Mitochondrial dysfunction is a core pathological event in AKI, characterized by excessive oxidative stress, energy metabolism failure and subsequent tubular epithelial cell apoptosis [22]. The present study demonstrates that limb RIPC effectively protects against renal IRI by enhancing functional mitophagy in renal tubular epithelial cells, in which SIRT3 acts as an indispensable molecular mediator. These findings clarify the mechanism underlying limb RIPC‐mediated renoprotection and establish a mechanistic link between RIPC and SIRT3‐dependent mitophagy, thereby further extending the theoretical framework of renoprotective strategies targeting mitochondrial quality control.

First, our results confirm that mitophagy serves as a necessary downstream effector of RIPC‐induced renoprotection. Consistent with Livingston et al. [6], who demonstrated that mitophagy is required for renal protection induced by conventional ischaemic preconditioning, our study further confirms that this mechanism is also applicable to limb RIPC, a more clinically accessible and translatable intervention. It is well known that excessive mitochondrial fission and impaired clearance of damaged mitochondria exacerbate renal IRI [23], and mitophagy represents a critical adaptive mechanism to maintain mitochondrial homeostasis. Our results showed that RIPC enhanced mitophagic activity in renal tubular cells, as indicated by increased LC3 II/I ratio, decreased p62 expression and increased autophagosome formation under electron microscopy. Moreover, the renoprotective effect of RIPC was largely abolished by the autophagy inhibitor 3‐MA, supporting that mitophagy activation is essential for RIPC to protect against renal IRI. Notably, our in vitro experiments verified that RIPC induces complete mitophagic flux (rather than autophagic blockage) via CQ inhibition assays, which complements the study by Fu et al. [24] that only observed RIPC‐induced mitochondrial function improvement without clarifying mitophagic flux integrity. This refinement highlights the specificity of RIPC‐regulated mitophagy in renal tubular cells.

Second, we identify SIRT3 as a key mediator linking RIPC to mitophagy. Previous studies have established the critical role of SIRT3 in renal IRI: Morigi et al. [25] demonstrated that SIRT3 protects against AKI by improving mitochondrial dynamics and maintaining mitochondrial homeostasis, while Zhao et al. [26] further confirmed that SIRT3‐dependent mitophagy serves as a core mechanism underlying renoprotection against both IRI and septic insults. Our study extends these important findings by demonstrating that RIPC upregulates SIRT3 expression to promote mitophagy—an effect that was abolished by SIRT3 knockout and effectively rescued by SIRT3 overexpression in vitro. This complete causal evidence chain supplements the study by Shen et al. [27], which focused on SIRT3‐mediated regulation of mitochondrial fusion but did not connect this pathway to RIPC. Moreover, our results align with the recent discovery by Deng et al. [28] that SIRT3 acts as a central hub coordinating mitophagy in stressed renal tubular cells, further reinforcing the notion that SIRT3 may serve as a promising therapeutic target for renal IRI.

Third, the Parkin‐mediated mitophagic pathway is a critical executive process for SIRT3‐dependent mitophagy in RIPC‐induced renoprotection. Parkin is a core E3 ubiquitin ligase that initiates mitochondrial mitophagy by mediating the ubiquitination of damaged mitochondrial proteins, and subsequent Parkin‐LC3B colocalization is a hallmark event for mitophagosome formation [29]. Accumulating evidence has confirmed that Parkin‐mediated mitophagy is a defensive mechanism against renal IRI, with impaired Parkin activity exacerbating tubular epithelial cell injury and mitochondrial dysfunction [30]. Our in vivo experiments demonstrated that IRI significantly reduced the colocalization of Parkin and LC3B in renal tubular epithelial mitochondria, while RIPC pretreatment markedly restored this key mitophagic event; importantly, the RIPC‐induced enhancement of Parkin‐LC3B colocalization was abrogated by 3‐MA, confirming that Parkin‐mediated mitophagosome formation is indispensable for RIPC‐induced functional mitophagy. These findings demonstrate that the Parkin‐mediated mitophagic pathway functions as a downstream executor of SIRT3‐dependent mitophagy in RIPC‐induced renoprotection.

Several limitations of this study should be acknowledged. First, 3‐MA is a non‐specific PI3K inhibitor that affects multiple cellular processes; thus, its effects cannot be solely attributed to mitophagy inhibition. Second, the precise molecular mechanism by which SIRT3 regulates Parkin‐mediated mitophagy remains to be further elucidated. Third, the use of rat RIPC plasma in in vitro experiments may limit direct clinical translation. Fourth, although renal IRI represents the core pathological basis of PCI‐AKI, we did not use a direct contrast‐induced AKI model; future studies in this model are warranted to strengthen clinical relevance. Finally, due to limited availability of SIRT3 knockout mice and adherence to the 3R principle, we did not include SIRT3 KO + Sham or SIRT3 KO + IRI groups. While the role of SIRT3 in renal IRI has been well documented, the absence of these groups limits formal interaction analysis. Future studies employing a complete 2 × 3 factorial design (WT/KO × Sham/IRI/RIPC + IRI) will be required to clarify genotype–treatment interactions.

Despite these limitations, our findings support the translational potential of limb RIPC—a non‐invasive, easily applicable intervention that can be implemented in patients undergoing PCI. Previous studies have shown that ischaemic preconditioning reduces contrast‐induced nephropathy [31], supporting the clinical relevance of our findings for PCI‐AKI prevention. Furthermore, the identification of SIRT3 and the Parkin‐mediated mitophagic pathway as core targets of RIPC provides potential molecular directions for developing combined therapeutic strategies. Future studies using human RIPC plasma and more selective mitophagy‐targeted interventions are warranted to validate and extend our findings.

## 5 Conclusions

This study demonstrates that RIPC alleviates renal IRI by activating SIRT3‐dependent mitophagic flux, with the Parkin‐mediated pathway serving as a critical downstream executor. Integrated in vivo and in vitro experiments—including SIRT3 knockout, siRNA knockdown, and overexpression—confirm the causal role of SIRT3 in RIPC‐induced renoprotection, while 3‐MA and chloroquine inhibition verify the integrity of mitophagic flux. As a non‐invasive and clinically feasible intervention, limb RIPC represents a translatable strategy for PCI‐AKI prevention. Furthermore, SIRT3 and the Parkin‐mediated mitophagic pathway may serve as potential molecular targets to enhance renoprotective efficacy, though the precise regulatory mechanism between SIRT3 and Parkin requires further investigation.

## Author Contributions

**Qian Cui:** conceptualization, methodology, writing – original draft. **Lina Zhao:** methodology, data curation, supervision, writing – original draft. **Wenjun Dai:** methodology, data curation. **Shuyan Sun:** data curation, methodology. **Yinan Li:** methodology, data curation. **Chenghui Zhou:** conceptualization, methodology, writing – review and editing. **Hanjun Pei:** conceptualization, methodology, supervision, writing – review and editing, project administration.

## Funding

This work was supported by the National Natural Science Foundation of China (No. 81760096).

## Ethics Statement

This study was approved by the Animal Ethics Committee of Baotou Medical College (Ethical Review No. 139 of 2020). All animal experiments were conducted in accordance with the ARRIVE guidelines and national animal experiment regulations. Since this study did not involve human subjects or tissue, informed consent to participate is not applicable.

## Consent

The authors have nothing to report.

## Conflicts of Interest

The authors declare that the research was conducted in the absence of any commercial or financial relationships that could be construed as a potential conflicts of interest.

## Data Availability

The datasets used and/or analysed during this study are available from the corresponding author (Hanjun Pei or Chenghui Zhou) on reasonable request. All materials (including reagents, cell lines and animal models) are commercially available or can be provided with proper authorization.

## References

[1] Z. Hassan , U. Kumari , U. Wasim , et al., “An Investigation of Contrast‐Induced Acute Kidney Injury in Patients Undergoing Percutaneous Coronary Intervention: A Cross‐Sectional Study From Pakistan,” Cureus 16, no. 2 (2024): e54725, 10.7759/cureus.54726. PMC10960921 38524020

[2] Y. Wang , J. Cai , C. Tang , and Z. Dong , “Mitophagy in Acute Kidney Injury and Kidney Repair,” Cells 9, no. 2 (2020): 338, 10.3390/cells9020338. PMC7072358 32024113

[3] Y. Li , Y. Wang , E. Kim , et al., “Bnip3 Mediates the Hypoxia‐Induced Inhibition on Mammalian Target of Rapamycin by Interacting With Rheb,” Journal of Biological Chemistry 282, no. 49 (2007): 35803–35813, 10.1074/jbc.M705231200. 17928295

[4] M. Zhan , I. M. Usman , L. Sun , and Y. S. Kanwar , “Disruption of Renal Tubular Mitochondrial Quality Control by Myo‐Inositol Oxygenase in Diabetic Kidney Disease,” Journal of the American Society of Nephrology 26, no. 6 (2015): 1304–1321, 10.1681/ASN.2014050457. PMC4446875 25270067

[5] C. Tang , H. Han , Z. Liu , et al., “Activation of BNIP3‐Mediated Mitophagy Protects Against Renal Ischemia‐Reperfusion Injury,” Cell Death & Disease 10, no. 9 (2019): 677, 10.1038/s41419-019-1899-0. PMC6742651 31515472

[6] M. J. Livingston , J. Wang , J. Zhou , et al., “Clearance of Damaged Mitochondria via Mitophagy Is Important to the Protective Effect of Ischemic Preconditioning in Kidneys,” Autophagy 15, no. 12 (2019): 2142–2162, 10.1080/15548627.2019.1615822. PMC6844514 31066324

[7] N. Tapuria , Y. Kumar , M. M. Habib , M. Abu Amara , A. M. Seifalian , and B. R. Davidson , “Remote Ischemic Preconditioning: A Novel Protective Method From Ischemia Reperfusion Injury—A Review,” Journal of Surgical Research 150, no. 2 (2008): 304–330, 10.1016/j.jss.2007.12.747. 19040966

[8] Y. Q. Long , X. M. Feng , X. S. Shan , et al., “Remote Ischemic Preconditioning Reduces Acute Kidney Injury After Cardiac Surgery: A Systematic Review and Meta‐Analysis of Randomized Controlled Trials,” Anesthesia & Analgesia 134, no. 3 (2022): 592–605, 10.1213/ANE.0000000000005804. 34748518

[9] W. N. Morley , A. M. Coates , and J. F. Burr , “Cardiac Autonomic Recovery Following Traditional and Augmented Remote Ischemic Preconditioning,” European Journal of Applied Physiology 121, no. 1 (2020): 265–277, 10.1007/s00421-020-04526-y. 33047259

[10] M. Billah , A. Ridiandries , U. K. Allahwala , et al., “Remote Ischemic Preconditioning Induces Cardioprotective Autophagy and Signals Through the IL‐6‐Dependent JAK‐STAT Pathway,” International Journal of Molecular Sciences 21, no. 5 (2020): 1695, 10.3390/ijms21051692. PMC7084188 32121587

[11] Y. Wang , J. Shen , X. Xiong , et al., “Remote Ischemic Preconditioning Protects Against Liver Ischemia‐Reperfusion Injury via Heme Oxygenase‐1‐Induced Autophagy,” PLoS One 9, no. 6 (2014): e98834, 10.1371/journal.pone.0098834. PMC4051645 24914543

[12] J. Su , T. Zhang , K. Wang , T. Zhu , and X. Li , “Autophagy Activation Contributes to the Neuroprotection of Remote Ischemic Perconditioning Against Focal Cerebral Ischemia in Rats,” Neurochemical Research 39, no. 11 (2014): 2068–2077, 10.1007/s11064-014-1396-x. 25082119

[13] W. Wei , L. Yang , B. Wang , et al., “Remote Ischemic Preconditioning Attenuates Mitochondrial Dysfunction and Ferroptosis of Tubular Epithelial Cells by Inhibiting NOX4‐ROS Signaling in Acute Kidney Injury,” International Journal of Biological Sciences 21, no. 5 (2025): 2313–2329, 10.7150/ijbs.105667. PMC11900797 40083709

[14] M. Gertz and C. Steegborn , “Using Mitochondrial Sirtuins as Drug Targets: Disease Implications and Available Compounds,” Cellular and Molecular Life Sciences: CMLS 73, no. 15 (2016): 2871–2896, 10.1007/s00018-016-2180-7. PMC11108305 27007507

[15] C. Gu , F. Kong , J. Zeng , X. Geng , Y. Sun , and X. Chen , “Remote Ischemic Preconditioning Protects Against Spinal Cord Ischemia‐Reperfusion Injury in Mice by Activating NMDAR/AMPK/PGC‐1α/SIRT3 Signaling,” Cell & Bioscience 13, no. 1 (2023): 57, 10.1186/s13578-023-00999-4. PMC10018930 36927808

[16] L. Perico , M. Morigi , and A. Benigni , “Mitochondrial Sirtuin 3 and Renal Diseases,” Nephron 134, no. 1 (2016): 14–19, 10.1159/000444370. 27362524

[17] W. Zhao , L. Zhang , R. Chen , et al., “SIRT3 Protects Against Acute Kidney Injury via AMPK/mTOR‐Regulated Autophagy,” Frontiers in Physiology 14, no. 9 (2018): 1526, 10.3389/fphys.2018.01526. PMC6246697 30487750

[18] C. Kilkenny , W. J. Browne , I. C. Cuthill , M. Emerson , and D. G. Altman , “Improving Bioscience Research Reporting: The ARRIVE Guidelines for Reporting Animal Research,” PLoS Biology 8, no. 6 (2010): e1000412, 10.1371/journal.pbio.1000412. PMC2893951 20613859

[19] N. Mizushima , A. Yamamoto , M. Matsui , T. Yoshimori , and Y. Ohsumi , “In Vivo Analysis of Autophagy in Response to Nutrient Starvation Using Transgenic Mice Expressing a Fluorescent Autophagosome Marker,” Molecular Biology of the Cell 15, no. 3 (2004): 1101–1111, 10.1091/mbc.e03-09-0704. PMC363084 14699058

[20] D. J. Klionsky , A. K. Abdel‐Aziz , S. Abdelfatah , et al., “Guidelines for the Use and Interpretation of Assays for Monitoring Autophagy (4th Edition),” Autophagy 17, no. 1 (2021): 1–382, 10.1080/15548627.2020.1797280. PMC7996087 33634751

[21] L. Zhou , R. Pinho , Y. Gu , and Z. Radak , “The Role of SIRT3 in Exercise and Aging,” Cells 11, no. 16 (2022): 2596, 10.3390/cells11162596. PMC9406297 36010672

[22] S. M. Parikh , Y. Yang , L. He , C. Tang , M. Zhan , and Z. Dong , “Mitochondrial Function and Disturbances in the Septic Kidney,” Seminars in Nephrology 35, no. 1 (2015): 108–119, 10.1016/j.semnephrol.2015.01.011. PMC4465453 25795504

[23] Z. Liu , H. Li , J. Su , et al., “Numb Depletion Promotes Drp1‐Mediated Mitochondrial Fission and Exacerbates Mitochondrial Fragmentation and Dysfunction in Acute Kidney Injury,” Antioxidants & Redox Signaling 30, no. 15 (2019): 1797–1816, 10.1089/ars.2017.7432. 29890853

[24] P. Fu and Y. L. Zhao , “Remote Ischemic Preconditioning Attenuates Mitochondrial Dysfunction and Ferroptosis of Tubular Epithelial Cells by Inhibiting NOX4‐ROS Signaling in Acute Kidney Injury,” International Journal of Biological Sciences 20, no. 5 (2025): 1589–1602. 10.7150/ijbs.105667 PMC11900797 40083709

[25] M. Morigi , L. Perico , C. Rota , et al., “Sirtuin 3–Dependent Mitochondrial Dynamic Improvements Protect Against Acute Kidney Injury,” Journal of Clinical Investigation 125, no. 2 (2015): 715–726, 10.1172/JCI77632. PMC4319434 25607838

[26] W. Zhao , M. Sui , R. Chen , et al., “SIRT3 Protects Kidneys From Ischemia‐Reperfusion Injury by Modulating the DRP1 Pathway to Induce Mitochondrial Autophagy,” Life Sciences 286 (2021): 120005. 10.1016/j.lfs.2021.120005 34606850

[27] L. Shen , Q. Zhang , S. Tu , and W. Qin , “SIRT3 Mediates Mitofusin 2 Ubiquitination and Degradation to Suppress Ischemia Reperfusion‐Induced Acute Kidney Injury,” Experimental Cell Research 407, no. 2 (2021): 112818. 10.1016/j.yexcr.2021.112861 34624325

[28] Z. Deng , M. He , H. Hu , et al., “Melatonin Attenuates Sepsis‐Induced Acute Kidney Injury by Promoting Mitophagy Through SIRT3‐Mediated TFAM Deacetylation,” Autophagy 20, no. 1 (2024): 151–165. 10.1080/15548627.2023.2252265 PMC10761103 37651673

[29] S. Pickles , P. Vigié , and R. J. Youle , “Mitophagy and Quality Control Mechanisms in Mitochondrial Maintenance,” Current Biology 28, no. 4 (2018): R170–R185. 10.1016/j.cub.2018.01.004 PMC7255410 29462587

[30] Q. Lin , S. Li , N. Jiang , et al., “PINK1‐Parkin Pathway of Mitophagy Protects Against Contrast‐Induced Acute Kidney Injury via Decreasing Mitochondrial ROS and NLRP3 Inflammasome Activation,” Redox Biology 26 (2019): 101254. 10.1016/j.redox.2019.101254 PMC6597739 31229841

[31] F. Er , A. M. Nia , H. Dopp , et al., “Ischemic Preconditioning for Prevention of Contrast Medium–Induced Nephropathy,” Circulation 126, no. 3 (2012): 296–303, 10.1161/CIRCULATIONAHA.112.096370. 22735306

